# Guidelines for the content of statistical analysis plans in clinical trials: protocol for an extension to cluster randomized trials

**DOI:** 10.1186/s13063-025-08756-3

**Published:** 2025-02-27

**Authors:** Karla Hemming, Jacqueline Y. Thompson, Richard L. Hooper, Obioha C. Ukoumunne, Fan Li, Agnes Caille, Brennan C. Kahan, Clemence Leyrat, Michael J. Grayling, Nuredin I. Mohammed, Jennifer A. Thompson, Bruno Giraudeau, Elizabeth L. Turner, Samuel I. Watson, Beatriz Goulão, Jessica Kasza, Andrew B. Forbes, Andrew J. Copas, Monica Taljaard

**Affiliations:** 1 Institute of Applied Health Research, University of Birmingham, Birmingham, UK; 2 Wolfson Institute of Population Health, Mary University of London, London, Queen UK; 3 NIHR Applied Research Collaboration South West Peninsula, University of Exeter, Exeter, UK; 4 Department of Biostatistics, Yale School of Public Health, New Haven, CT USA; 5 Université de Tours, Université de Nantes, INSERM, SPHERE U1246 Tours, France; 6 INSERM CIC1415, CHRU de Tours, Tours, France; 7 MRC Clinical Trials Unit at UCL, London, UK; 8 Department of Medical Statistics, London School of Hygiene and Tropical Medicine, London, UK; 9 Janssen R and D, High Wycombe, London, UK; 10 MRC Unit The Gambia at the London, School of Hygiene and Tropical Medicine, Banjul, The Gambia; 11 Department of Infectious Diseases and International Health, London, School of Hygiene and Tropical Medicine , London, UK; 12 Department of Biostatistics and Bioinformatics and Duke Global Health Institute, Duke University, Durham, NC 27705 USA; 13 Health Services Research Unit, University of Aberdeen, Aberdeen, UK; 14 School of Public Health and Preventive Medicine, Monash University, Victoria, Melbourne, Australia; 15 MRC Clinical Trials Unit, University College London, London, UK; 16 Clinical Epidemiology Program, Ottawa Hospital Research Institute, 1053 Carling Avenue, Ottawa, ON Canada; 17 School of Epidemiology, Public Health and Preventive Medicine, University of Ottawa, Ottawa, Canada

**Keywords:** Cluster randomized trials, Group randomization, Statistical analysis plans, Analysis plans, Guidelines

## Abstract

**Background:**

Guidance exists to inform the content of statistical analysis plans in clinical trials. Though not explicitly stated, this guidance is generally focused on clinical trials in which the randomization units are individual patients and not groups of patients. There are critical considerations for the analysis of cluster randomized trials, such as accounting for clustering, the risk of imbalances between the arms due to post-randomization recruitment, and the need to use small sample corrections when the number of clusters is small.

**Methods:**

This paper outlines the protocol for the development of a set of reporting guidelines for the content of statistical analysis plans for cluster randomized trials (including variations such as the stepped wedge cluster randomized trial and other cluster cross-over designs) by extending the minimum reporting analysis requirements as previously defined for individually randomized trials to cluster randomized trials. The guideline will be developed using a consensus-based approach, modifying existing reporting items from the guideline for individually randomized trials and extending to include new items.

**Discussion:**

The guideline will be developed so it can be used independently of the guideline for individually randomized designs. The consensus guidelines will be published in an open-access journal, including key guidance as well as exploration and elaboration.

## Introduction

The “Guideline for the Content of Statistical Analysis Plans in Clinical Trials,” published in 2017 and referred to here as the 2017 SAP guidelines, outlines a minimum set of 32 items that should be included in the statistical analysis plans (SAPs) for clinical trials [1]. To accommodate early phase (phase I and non-randomized phase II) clinical trials, the guideline was extended in 2022, modifying 25 of the initial items and adding a further 11 items [2]. Although it is not explicitly stated, these guidelines are generally focused on clinical trials in which the units of randomization are individual patients.

The use of cluster randomization, in which the unit of randomization is a cluster rather than an individual, has been steadily increasing over the past decades [3]. Common reasons for cluster randomization include the evaluation of complex interventions when it is infeasible to randomize individuals, the need to simplify trial processes, the need to avoid within-cluster contamination, or when the objectives pertain to the cluster level [4–7]. Cluster randomized trials (CRTs) are known to have many additional complexities in their design, execution, and analysis, compared to individually randomized designs [5, 6, 8, 9]. They also have unique reporting requirements: the CONSORT guidelines for reporting parallel arm randomized trials were extended to cluster randomized designs in 2004 and updated in 2012 [10]. Additional extensions were later developed to accommodate novel cluster-randomized designs: specifically, the stepped wedge cluster randomized trial (SW-CRT) in 2018 [11, 12] and the cluster randomized cross-over design in 2024 [13].

Some of the key analytical considerations for cluster randomized trials are outlined in Table 1.. Most importantly, clustering should always be allowed for in the statistical analysis using one of several available methods [14]. Additionally, when the number of clusters is small—less than about 40—a “small sample correction” is usually needed to avoid biased estimation of the standard errors [14]. Furthermore, cluster randomized trials often need to recruit participants post-randomization, which increases the risk of imbalances between the arms at baseline [15–18]. This may then necessitate statistical adjustment in the primary analysis, for example, using direct covariate adjustment or a propensity score approach [19, 20]. There are numerous other complexities, including, according to the CONSORT extension for CRTs, the need to have clarity around “specific objectives and hypotheses and whether they pertain to the individual level, the cluster level, or both” [10, 21]; whether interest is in an average treatment effect across individuals or average across clusters [22]; and whether covariates are defined at cluster-level, individual level, or both [21]. Existing guidance for elements to report in SAPs is insufficient to cover these and other complexities; explicit guidelines are required for CRTs.

**Table 1:** Preliminary considerations of importance when constructing statistical analysis plans for cluster randomized trials

Issue	Elaboration	Change	Potential items of relevance (2017 SAP guidance)
Terminology – participant recruitment	Many cluster randomized trials do not have direct participant recruitment (i.e., asking participants and obtaining their consent for trial participation) [Parker 2021] Thus, whilst many patient randomized trials describe and refer to participant recruitment, this is not always an appropriate term in cluster randomized trials	Careful use of the terminology “recruitment”, changing to “identification of participants” where appropriate	Throughout
Terminology – identification and recruitment bias	Cluster trials sometimes do include participant recruitment, and this identification and recruitment of participants can occur after clusters have been randomized. Where this is the case, it is important that this is clearly reported (as it might be associated with differential recruitment across treatment conditions) [Eldridge 2009] Following the RoB2 tool guideline, we refer to this as identification and recruitment bias, not selection bias		Item 10b Item 21 Item 23
Presentation of cluster and individual-level baseline characteristics	In CRTs, when presenting the characteristics across study arms at baseline, there are two levels of characteristics to be presented – the cluster-level and individual-level characteristics. Most probably agree that no statistical tests should be done regarding the cluster-level characteristics, which are the randomized units. Things are not so clear for individual-level baseline characteristics	Clarify that the characteristics for presentation in the baseline table should include both cluster and individual-level characteristics. Confirm that no statistical testing should be carried out on the cluster-level characteristics	Item 25a
Estimands	It is important in all trials to be clear about what it is we are trying to estimate (i.e. the estimand) [Kahan 2023]. In CRTs, there are additional things we need to consider when defining an estimand, for instance, whether interest lies in the average effect over clusters or individuals; whether interest lies in marginal or cluster-specific effects; or how issues like non-adherence at the	Clarify target estimand(s) for each outcome; ensure analysis approach aligns with target estimand; and state the key assumptions the analysis approach requires to be unbiased for the target estimand	Item 8 (study objectives) Potential new Item 26 (see SAP guidance for early phase trials where a new item for estimands was introduced)
	cluster level are handled. These issues matter because the value of these different estimands may differ in magnitude, and hence offer different conclusions about a treatment’s benefits or harms [Kahan 2023]. For example, the CONSORT for CRTs asks researchers to outline whether the objectives pertain to the individual or cluster levels [Campbell 2012]		Item 27 under analysis
Clustering	The analysis of individually randomized trials assumes independence of observations. However, when treatment assignment depends on the cluster, analysis approaches used for individually randomized designs are generally not appropriate There are several approaches that can accommodate the clustered nature of the data, including: a cluster-level analysis, generalised linear mixed models, and generalised estimating equations [Turner 2021]. Less commonly used alternatives include the use of cluster-robust standard errors. More complicated correlation assumptions may be required in multiple-period designs It is also important to report measures of clustering, such as the intra-cluster correlation coefficient (ICC) – or an equivalent, such as the coefficient of variation of outcomes across clusters. Information is also required on how this clustering will be estimated (along with confidence interval calculation); what scale it will be reported on; and whether it will be estimated separately in each arm or jointly across arms	How clustering will be allowed for in the analysis For multiple-period designs, consideration of more complex correlation structures and adjustment for time effects	Item 27a (analysis) New item for the ICC
Small sample corrections	Common approaches for constructing confidence intervals, and estimating pvalues make asymptotic assumptions that are only valid in large samples. In CRTs, because there are typically fewer than about 40 clusters, these approximations are often invalid [Turner 2021]. Rather, some correction to the standard error is required, or the use a tdistribution rather than the normal distribution. When using the t-	Allowance for small sample corrections in the analysis	Item 27a
	distribution, the appropriate choice for the degrees of freedom can be challenging		
Non-convergence	The 2017 SAP guidance included specification of alternative analysis methods to be used if distributional assumptions do not hold. In CRTs, where multi-level models are commonly used, non-convergence is also a particular concern. It is important to consider in advance how this will be handled	Model analysis back up plan in case of nonconvergence	Item 27d
Covariate adjustment	In individually randomized trials, covariate adjustment can improve statistical power [Sullivan 2024]. In CRTs, covariate adjustment can have another very important role, especially when there is post-randomization identification of participants [Parker 2012; Parker 2022]. Some important considerations are: whether the covariates will be prespecified, their functional form, whether they will be included as individual-level or cluster-level; how this will be implemented (direct covariate adjustment, or propensity scores) and whether a covariate-adjusted analysis will form the primary analysis	Clarity around features of covariate adjustment	Item 27b New item for estimands
**Issues which likely overlap with items for future consideration in the main guidance**			
Protocol deviations	The 2017 SAP guideline includes reporting on protocol adherence, for example, compliance with pill intake Many CRTs evaluate implementation strategies or behaviour change interventions. Here, non-adherence can be an outcome in itself, forming part of a process evaluation or implementation evaluation. The language used to describe these terms, along with how the focus can be on evaluation rather than descriptive summaries, might need further consideration in some SAPs. This issue is not unique to CRTS but rather is of relevance to pragmatic trials more generally Furthermore, in some CRTs, nonadherence can occur at either the individual level or the cluster level, and it is useful to understand and report both	Protocol deviations were replaced with process evaluation	Item 19a and 19b
	For stepped wedge trials, non-adherence can also include departures from the timing of the randomization schedule However, whilst replacing the item “protocol deviations” with a “process evaluation” might be one option, it will also be important to retain an item which relates to non-adherence/intercurrent events		
Internal pilots	Whilst the 2017 SAP guideline has an item on reporting interim analyses (item 13), it does not mention internal pilots. It might be that the sorts of trials the guideline was targeted at (regulatory) do not often have internal pilot studies. Nevertheless, they are commonly encountered in CRTs and other pragmatic trials The wording under the item about interim analysis could be expanded to include internal pilots (which are essentially a form of stopping rules) There is also some careful wording about blinding in the 2017 SAP guideline, which is likely to be more important for regulatory trials	Discussion of internal pilot data	Item 13

Here, we describe our protocol to develop an extension to the guidelines for the reporting of SAPs to cluster randomized trials. Our planned extension will cover conventional parallel arm CRT designs, as well as multi-period or longitudinal designs, such as the stepped wedge cluster randomized trial and the cluster randomized cross-over design. We plan to develop a standalone SAP guidance document that can be used independently of other statistical analysis plan guidelines.

### Aims and objectives

The overarching aim of this work is to produce a standalone extension of the 2017 guideline for reporting of statistical analysis plans for cluster randomized trials with elaboration and explanation. To accomplish this goal, our specific objectives are to:

1. Search the peer-reviewed literature and the EQUATOR website to identify specific guidelines and recommendations relevant to the reporting or methodology of cluster randomized trials.
2. Review a sample of available (published or unpublished) SAPS for CRTs to identify current practices in reporting.
3. Develop an initial draft set of items based on the results from objective 1 and with input from an expert working group, supplemented with examples from the SAPs identified in objective 2.
4. Implement a Delphi survey, starting with the initial draft set of items from objective 3, with two or three rounds of surveys of a wider group of stakeholders, to obtain a draft of the SAP guideline extension.
5. Conduct a consensus meeting with the working group to produce the final SAP guideline extension, along with explanation, elaboration, and examples.
6. Conduct a pilot test of the guideline by using it to inform the development of SAPs for cluster randomized trials.

## Methods

Our protocol development was informed by the recommendations for developing reporting guidelines [13, 23]. This protocol was registered on the Enhancing the QUAlity and Transparency Of health Research (EQUATOR) website in (May 2023). The outlined protocol was presented to members of the core working group (defined below) in June 2023 and to the wider working group (defined below) in May 2024 for feedback and agreement.

### Objective 1: Search the peer-reviewed literature and the EQUATOR website to identify specific guidelines and recommendations relevant to the reporting or methodology of cluster randomized trials

We will review the EQUATOR network repository to identify any existing reporting guidelines for SAPs or trial methods relevant to this extension. We conducted an initial search on 31 March 2023 and identified six relevant documents (Table 2). This search will be updated when work on the guideline development starts.

**Table 2:** Identified relevant guidelines to either cluster randomized trials or statistical analysis plans

Title	Reference	Link
2017 SAP guidelines	[Gamble 2017]	http://lctc.org.uk/SAP-Statement
SAP for early phase trials	[Homer 2022]	https://www.bmj.com/content/376/bmj-2021068177
CONSORT for cluster trials	[Campbell 2012]	https://www.equator-network.org/reportingguidelines/consort-cluster/
CONSORT for SW-CRTs	[Hemming 2018]	https://www.equator-network.org/reportingguidelines/reporting-of-stepped-wedge-clusterrandomised-trials-extension-of-the-consort-2010statement-with-explanation-and-elaboration/
CONSORT for cluster crossover trials	[McKenzie 2024]	Submitted for publication April 2024 https://doi.org/10.31222/osf.io/psemy
RoB2 Risk of Bias tool for cluster randomized trials	[Sterne 2019]	https://methods.cochrane.org/bias/resources/rob- 2-revised-cochrane-risk-bias-tool-randomized-trials

To supplement the search of EQUATOR, we will consult key methodological papers to identify relevant reporting considerations for CRTs that are not covered in any existing guidelines. For example, the importance of clearly reporting whether interest lies in the marginal vs. cluster-specific effect was recently identified in the methodological literature. Key methodological papers will be identified through journal searches, through consultation with the expert working group (many of whom are statisticians conducting methodological research relevant to CRTs), and during the Delphi process.

#### Objective 2: Review a sample of available (published or unpublished) SAPS for CRTs to identify current practices in reporting

Identifying a representative sample of SAPs is challenging. Firstly, few investigators publish their SAPs (< 1%) in peer-reviewed journals; secondly, the response rate in a survey of investigators requesting the sharing of their unpublished SAPs would likely yield a low response rate (~ 8%) [24]. We plan to conduct a comprehensive electronic search to identify published SAPs. We will also supplement this search with SAPs previously identified as part of an independent systematic review of primary reports of CRTs, which was undertaken as part of a different project [25]. This was an efficient way to identify unpublished SAPs that had been uploaded as supplementary material to the primary trial publication.

The search for published SAPs will be conducted in MEDLINE via PubMed (Table 3). The search terms will be based on previously published electronic search strategies [26], will use several synonyms to describe the cluster design, and will consider alternative spelling (e.g., cluster randomiz(s)ed trials). We will aim to identify SAPs for any type of cluster randomized trial (parallel or multi-period). We will exclude SAPs related solely to feasibility studies. Note that this search will necessarily identify fully published standalone SAPs with their own DOI (but not SAPs uploaded as supplementary material to a primary trial report). An exploratory search was implemented on 30 May 2023 and identified 53 results (before screening for inclusion and exclusion criteria) and 23 that met our inclusion criteria.

**Table 3:** PubMed search criteria for identification of peer-reviewed standalone statistical analysis plans

Search (#)	Search terms
#1	(cluster-randomi*[tiab] OR “cluster randomized”[tiab] OR “cluster randomized”[tiab] OR “cluster randomization”[tiab] OR “cluster randomisation”[tiab])
#2	(group-randomi*[tiab] OR “group randomized”[tiab] OR “group randomized”[tiab] OR “group randomization”[tiab] OR “group randomisation”[tiab])
#3	“statistical analysis plan”[tiab]
#4	#1 OR #2 AND #3
#5	#4 NOT(animals [mh] NOT (humans [mh]))
#6	#5 (("2015/01/01"[Date—Publication]: "2023/05/30"[Date—Publication]))

To supplement our search, we will include SAPs identified as part of an independent systematic review of primary reports of CRTs taking place exclusively in low-income, lower-middle-income countries (LMICs) [25]. The methods and results for the review are reported elsewhere but in brief: a previously developed and validated electronic search filter for CRTs was implemented in combination with Cochrane’s highly sensitive and precision maximizing strategy for randomized controlled trials [27–29] together with a complete list of countries classified as low-income, lower-middle-income, or upper-middle-income according to the World Bank’s classification of countries by income [30]. The search was implemented in Ovid MEDLINE on August 17th, 2022, and was further limited to articles published in the English language between January 1st, 2017, and the search date. The search identified around 800 eligible trials. Each trial report was reviewed to identify references to any published or unpublished SAP. This sample will be used to identify published SAPs missed by our search above (expected to be a limited number only) and publicly available SAPs that have not been published with their own unique DOI.

The identified sample of published standalone and non-standalone SAPs for CRTs will be used to:

1. Identify lead statisticians and other relevant team members (e.g., the corresponding author) to include in the Delphi survey;
2. Identify examples of good practice to be used in the explanation and elaboration document;
3. Describe current practices in the reporting of SAPs for CRTs;
4. Inform the development of a draft set of reporting items for the planned extension.

### Objective 3: Develop a first draft of the guideline (based on the guidelines identified in 1 above, modified by an expert working group, and supplemented with examples from the SAPs identified in 2 above) that will form the initial Delphi content

An initial draft set of items will be produced by a core working group (KH, MT, RLH, AJC, ABF, BG, BCK, JYT). This initial draft will be informed by the results from objective 1. It will consider any necessary modifications to the existing SAP guidance document to accommodate CRTs (see Table 1. for a summary of potentially relevant modifications for CRTs) as well as the extension for early phase trials (see Table 4 for a summary of potentially relevant modifications made to the early phase trial SAPs guideline). We will also consult reporting guidelines for CRTs (CONSORT statements) to ensure consistency in the use of wording and streamlining across reporting guidelines (Table 1.). The SAPs for CRTs identified in objective 2 will be reviewed to identify any further items of potential consideration and case studies for inclusion in the elaboration and explanation.

**Table 4:** Major modifications between the 2017 SAP guideline and the SAP guideline for early-phase trials

Modification and Item number (early phase guideline)	Detail (copied verbatim)	Relevance to CRTs
Statistical design (Items 9a-9e)	Increased details regarding statistical design methodology, and model choice where appropriate	Not considered relevant to CRTs (in early phase trials, this is about whether it is a dose escalation trial or single arm design)
Estimands (Items 26b, 26c, 26d, 26e, 27a, 27e, 27f)	Update of outcome definitions to include the definition of estimands in line with the principles outlined in ICH E9 (R1)	To include for consideration in CRT guideline
Simulation studies (Items 31, 33)	Inclusion of simulation reports incorporating operating characteristics, to justify statistical design or sample size where applicable	Less relevant for CRTs, which tend to perform sample size calculation via analytical formulae (as opposed to phase I designs where that is not typically possible)
Code (Items 30, 35)	Inclusion of code required for novel methodology	To include for consideration in CRT guideline
Dose transition pathways (Item 9d)	Inclusion of dose transition pathways, where appropriate	Not considered relevant to CRTs
Bayesian approaches (Items 12, 18)	Amendments to wording to be more neutral to both frequentist and Bayesian methodology, to reflect that some early phase clinical trial designs, particularly phase I, are underpinned by Bayesian methods	Unlikely to be of important consideration for CRTs (although the analysis and interpretation of statistical findings using a Bayesian approach is a very valid alternative to the frequentist approach in CRTs)

The initial draft set of items will then be distributed for review and input to the wider expert working group (all co-authors of this protocol). The working group includes broad representation from countries and regions that conduct the majority of CRTs (Europe, Australia, USA, and Canada) and includes statisticians from both academia and the pharmaceutical industry, statisticians who were born, work or live in LMICs, and funders and journal editors. The initial draft set of items will be modified in response to their comments and will then be used to prepare a Delphi survey [31].

#### Objective 4: Conduct two or three rounds of a Delphi survey to elicit the views of wider stakeholders to obtain an initial consensus for the guideline.

All of the members of the core working group have experience and skills of designing, running, and analyzing trials, predominantly (but not exclusively) CRTs; in conducting Delphi surveys and consensus meetings. We will draw on this experience but also look to ensure this process is carried out to an appropriate high standard, learning from our past experiences, and leveraging the development of the methodology of the process itself. In advance of starting the procedure, we will create a study plan, including invitation letters, design the survey tool, pilot test all elements, consider approaches to ensure anonymity, and define the level of consensus in advance. The process will be subject to University ethical approval, as is standard with any user engagement project.

##### Selection of Delphi survey participants

We will use a mixture of purposive sampling and snowball sampling to identify Delphi survey participants. We will aim to include a similar range of expertise and perspectives as those included in the wider working group but will also target chief investigators. To identify potential participants, we will use distribution lists obtained from the UK Clinical Research Collaboration registered Clinical Trial Units (UKCRC CTUs), members of the Canadian Network for Statistical Training in Trials (CANSTAT), members of the Statistical Society of Australia (SSA), members of the Australian Clinical Trials Alliance (ACTA), and the Global Health Network. Additional avenues of dissemination will include:

1. The circulation list for the Delphi survey used for the CONSORT extension for SW-CRTs;
2. Corresponding authors of SAPs identified in objective 2; and
3. Dissemination by the full working group.

We will not use wide circulation on social media so as to limit the participants to those with known expertise. The survey will include questions on the level of expertise in CRTs, academic area of expertise, predominate clinical areas of work, as well as region of work.

##### Consent and expectations of survey participants

People will be asked by email invitation if they wish to participate in the study. They will be provided with a participant information sheet. Participants will be asked if they consent to participation when they open the survey. Anyone who does not wish to participate can also decline participation by not completing the survey. Participants in the Delphi exercise will be offered the opportunity to be acknowledged by name and affiliation in the resulting publications. Participants will be asked for their emails if they wish to be acknowledged in the publication. To maintain the engagement of those who have committed, we will provide clear information on expectations and only obtain opinions on a clearly agreed set of study items (to prevent the survey from being too long). To balance the number of rounds to obtain consensus and questionnaire fatigue, we intend to use between 2 and 3 rounds, which has been found to be optimal [31]. Round one will include an open-ended set of questions so as to ensure all opinions are gathered.

##### Delphi survey structure

The survey will proceed through each of the SAP items in turn. Participants will be provided with the proposed corresponding item for the cluster extension statement. For items where a modification is being proposed, participants will be asked to indicate whether they agree or disagree with this suggested modification of the item. Participants will be able to provide comments on the proposed modification (for example, suggest alternative wording). For items where no modification is suggested, participants will be asked to indicate if they agree or disagree with having no modification, and they can make suggestions for any modifications. An example of the proposed format is included in Table 5 (anticipated minor change) and Table 6 (anticipated new item).

**Table 5:** Example of proposed format for Delphi survey for Item 10 (minor change anticipated)

**Standard SAP item:**	Randomization details, e.g., whether any minimization or stratification occurred (including stratifying factors used or the location of that information if it is not held within the SAP)
**Proposed SAP for cluster extension*** (change highlighted in red italics)***:**	Randomization details, *including whether any restricted randomization was used, e.g., covariate-constrained randomization or stratification* (including stratifying factors used or the location of that information if it is not held within the SAP)
**Justification for proposed change:**	Most CRTs use some form of restricted randomization [Turner 2021]. However, the types of restricted randomization methods used in CRTs are different from those commonly used in individually randomized designs. For example, minimization is commonly used in individually randomized designs but not in CRTs, where stratification and covariate-constrained randomization are more commonly used. The wording has thus been modified to reflect the types of restricted randomization commonly used in CRTs
**Example (with citation details)**	“The details on the randomization procedures are in the Protocol [cited]. In brief, covariate-constrained randomization, stratified by historic transplant center referral patterns, was used to allocate the 26 CKD programs (1:1) to the intervention arm or the usualcare arm” *This example would ideally have had more information on factors included in the restricted randomization. The SAPs identified in the systematic reviews will hopefully identify better examples*
**Reference**	Dixon SN, Naylor KL, Yohanna S, McKenzie S, Belenko D, Blake PG, Coghlan C, Cooper R, Elliott L, Getchell L, Ki V, Mucsi I, Nesrallah G, Patzer RE, Presseau J, Reich M, Sontrop JM, Treleaven D, Waterman AD, Zaltzman J, Garg AX. Enhance Access to Kidney Transplantation and Living Kidney Donation (EnAKT LKD): Statistical Analysis Plan of a Registry-Based, Cluster-Randomized Clinical Trial. Can J Kidney Health Dis. 2022 Nov PMID: 36,438,439; PMCID: PMC9693773
**Proposed explanation and elaboration text**	Most CRTs use some form of restricted randomization [Turner 2021]. Restricted randomization methods can enhance the credibility of the trial results by protecting against imbalances in cluster and participant characteristics and can also improve statistical power [Ivers 2012] Restricted randomization methods use either clusterlevel characteristics or cluster-level summaries of individual-level characteristics (e.g. cluster-level mean of primary outcome from a baseline period) There are a number of different approaches for restricted randomization in CRTs, including
	stratification, covariate-constrained randomization and pair matching. Blocking can help prevent large imbalances in the number of clusters allocated to each arm As is the case with individual randomization, when restricted randomization has been used, the analysis should adjust for the covariates used in the randomization to ensure nominal type I errors and improve power [Li 2017]
**Do you agree with the proposed modification to the wording for this item?**	
	Agree
	Disagree
	Uncertain
**Comments on this suggested modification**

**Table 6:** Example of proposed format for Delphi survey for Item 10b (new item anticipated)

**Item name**	Post randomization recruitment
**Standard SAP item:**	None
**Proposed SAP for cluster extension*** (new item)***:**	Information on the timing of randomization with respect to any participant recruitment
**Justification for proposed change:**	Post-randomization recruitment can lead to recruitment bias. In CRTs with post-randomization recruitment, there may be even more reasons to consider covariate adjustment very carefully
**Example (with citation details)**	The ALAPAGE study includes a schematic representation of the study design, which clearly shows how participants were recruited after randomization. The analysis section of the SAP includes a plan to adjust for covariates to mitigate any impact of recruitment bias across arms *Again, ideally, this example would report text in the manuscript that described these recruitment procedures. SAPs identified from the systematic review will hopefully include better reporting styles*
**Reference**	Bocquier A, Jacquemot AF, Dubois C, Tréhard H, Cogordan C, Maradan G, Cortaredona S, Fressard L, Davin-Casalena B, Vinet A, Verger P, Darmon N; ALAPAGE Study Group; Arquier V, Briclot G, Chamla R, Cousson-Gélie F, Danthony S, Delrieu K, Dessirier J, Féart C, Fusinati C, Gazan R, Gibert M, Lamiraud V, Maillot M, Nadal D, Trotta C, Verger EO, Viriot V. Study protocol for a pragmatic cluster randomized controlled trial to improve dietary diversity and physical fitness among older people who live at home (the "ALAPAGE study"). BMC Geriatr. 2022 Aug 4;22(1):643. https://doi.org/10.1186/s12877-022-03260-8. PMID: 35,927,684; PMCID: PMC9351201
**Proposed explanation and elaboration text**	In individually randomized trials, individuals are randomized to the treatment or control condition after they have agreed to participate. In CRTs, individual participants may be recruited or identified after clusters have been randomized [Eldridge 2009; Parker 2021; Parker 2022]. This can lead to differential inclusion of participants across treatment conditions, leading to an important source of bias [Easter 2022]
	Whether participants were recruited postrandomization can help justify whether the primary analysis should be adjusted for additional covariates (other than those included in any restricted randomization). Covariates of importance will be not only those that are associated with the outcome, but also those that are predictive of identification and recruitment into the trial
**Do you agree with the proposed modification to the wording for this item?**	
	Agree
	Disagree
	Uncertain
**Comments on this suggested modification**	

##### Analysis of the Delphi rounds

The Delphi exercise will be carried out to obtain a preliminary consensus. This will be carried out in an iterative process: proposing item modifications, asking for opinions on the modifications, modifying items in response, and feeding back changes. These preliminary agreed set of items will be taken forward to a consensus meeting (see objective 5). The Delphi survey will be carried out electronically. All responses will be anonymous. To define consensus, we will use percentage agreements (above 90% will be taken as agreement) and consistency of responses between rounds.

1. Delphi preliminary round
  The first draft of the set of items developed by the core members of the working group will form the initial preliminary Delphi round. This preliminary round will both act as a pilot test of the Delphi process and implementation (i.e., testing out the mechanics of the survey) as well as acting as a way in which all members of the expert working group can provide their opinions on the initial items (as proposed by the core working group). The revised content from this preliminary round will go forward to Delphi Round One.
2. Delphi Round One
  Respondents to the Delphi survey will be asked to indicate their level of agreement with each proposed item.
  Respondents will be invited to provide text-based comments on the terminology and wording used, as well as to suggest example trials for case studies.
3. Scoring and synthesis of Round One
  Scoring and synthesis of Round One
  Any text-based comments will also be synthesized.
4. Delphi Round Two
  The percentage agreement and responses will be fed back to participants along with the initial items. Any items clearly identified in Round One as having met a consensus (see above) will be removed from Round Two.
  Respondents to the Delphi survey will be again asked to indicate their level of agreement with each proposed item.
  Respondents will again be invited to provide text-based comments on the terminology and wording used; and suggest case studies.
5. Scoring of Round Two
  Round Two will be scored and synthesized in the same way as Round One. These modified items and scores will be taken to the consensus workshop.
6. A third round will be included if deemed appropriate by the core working group

#### Feedback of study results to participants of the Delphi survey

Participants will be asked during the survey if they would like to receive a copy of the final published paper. Participants will also be asked if they would like to be acknowledged for their replies in any reports or publications arising from this work. Any participants responding positively to either of these questions will be asked to provide their name, affiliation, and email address. Individual responses will not be publicly linked with their name.

#### Objective 5: Conduct a consensus meeting with the expert working group to produce the final guideline, along with an explanation and elaboration with examples

Members of the expert working group (i.e., all authors of this protocol) will be invited to participate in a consensus workshop. This workshop will take place as a face-to-face meeting, with a virtual option to allow greater flexibility and the widest geographical representation. All items identified by the Delphi exercise will be reviewed by this group. Those items for which there is contention over either the inclusion of the item or the wording of the item will be discussed in more detail. To this end, the findings from the Delphi exercise will be reported in a fair and un-prejudiced way (possibly by an independent person), and the meeting will be recorded. Any items under discussion will be scored and revised as described for the electronic Delphi exercise. If the expert working group cannot reach a consensus for any of the items, then this will be reported openly as such in the reporting guideline.

In the consensus meeting, we will use methods to mitigate the impact of dominant personalities and minimize group pressure. In our past experience, the key to this was an experienced chair—not necessarily a subject expert and unlikely to be a member of the core or expert working group.

#### Objective 6: Conduct a critical review and piloting of the guideline by using it to inform the development of statistical analysis plans for cluster randomized trials.

Members of the core working group will undertake critical review and piloting, which will consist of incorporating the guideline modifications into existing SAP templates (developed in accordance with the original guideline content for SAPs). At least one UK CTU (Birmingham CTU) will implement these, where the template acts as a quality control document. Feedback will be obtained, and updates on the wording of the elaboration and explanation will be considered.

##### Patient and public involvement (PPI)

Patient involvement in statistical aspects of clinical trials is an area of evolving research with very limited consideration to date [32]. SAPs are ultimately about ensuring trial analyses are conducted in a transparent and reproducible way, that answers relevant questions to patients, healthcare professionals, and other relevant stakeholders. Early research suggests that patients might have a role in the presentation of results for trial participants and the interpretation and presentation of findings more widely [33, 34]. We therefore include academics in our expert working group who have experience in understanding how patients might be involved in SAPs (BPG) [34].

## Discussion

The consensus guidelines will be written up as a peer-reviewed document, and published in an open-access journal, including key guidance as well as exploration and elaboration. This guidance will be disseminated using a variety of means, including but not limited to the EQUATOR website and an existing website designed to support those conducting CRTs and SW-CRTs: https://clusterrandomizedtrials.qmul.ac.uk and be presented at the annual meeting of “Current Developments in Cluster Randomized Trials and Stepped Wedge Designs” which attracts in the region of 100 cluster trialists annually. While we intend that this guideline is primarily concerned with the reporting quality of SAPs, we nonetheless will include some guidance on good practice within the explanation and elaboration.

### Limitations

Our study has several limitations. One limitation pertains to the review of available SAPs. The publication of SAPs can take different forms [35], for example, reported within the study protocol, as an appendix to the study protocol or final trial report, or as a separate peer-reviewed publication. SAPs published as separate standalone documents will have undergone peer review and may be better exemplars of good reporting. Our review of SAPs will include both standalone peer-reviewed publications and SAPs available as supplementary material to final trial reports or protocols, which may reflect a high degree of variability in reporting practices. We will not contact investigators directly to obtain SAPs and thus, our sample may include SAPs of higher quality. Others have identified low response rates when directly contacting investigators for unpublished protocols and SAPs [24].

Another limitation is that our review of SAPs will contain proportionately more CRTs conducted in LMICs than compared to other regions. There are likely to be a number of differences between CRTs conducted in a clinical setting compared to those CRTs conducted in the context of interventional research for population health, likely to be captured in the review of CRTs conducted in LMICs. In the latter, we often randomize geographical areas, as well as facilities (e.g., health centers), we do not always know who exactly the members of the clusters are, and sometimes, there is no recruitment or no consent taken from individual participants.

Some limitations also pertain to the Delphi survey, which does not use a random sample of participants. This is in line with previous approaches to developing guidelines for SAPs. While we have not restricted inclusion in the survey to statisticians, our targeted approach means that statisticians are likely to be the dominant responders. The SAP is a technical document, and it is unlikely that a non-statistician can contribute over and above any contribution they make to the protocol. However, we will include academics in our expert group who have experience understanding how patients might be involved in SAPs, and we will have a wider consultation about whether patients might be involved in this project.

### Estimands

The importance of specifying the target estimand has seen increasing prominence in recent years [22]. The field of estimands in CRTs needs further development [5]. In CRTs, we need to be very clear about what it is we are trying to estimate. Is it, for example, some kind of average of the effect over the randomized population, and in this case, what sampling process is implicit in this “averaging” [22]. Indeed, the adaptation of the guidance for reporting of SAPs to early phase trials incorporated the estimand framework [2]. In this guideline, we do propose to include the concept of the estimand, and it is expected that this part of the guideline will be one of the parts that require the most careful consideration.

## Summary

Similar to the process utilized by Gamble et al. [1], we have devised a process to enable us to develop analogous guidance for the development and reporting of statistical analysis plans for cluster randomized trials. This initial guidance should, in due course, improve the quality of the reporting of statistical analyses of CRTs as well as having the potential to improve the quality of the conduct of these analyses.

## Abbreviations

ACTA: Australian Clinical Trials Alliance
CANSTAT: Canadian Network for Statistical Training in Trials
CONSORT: Consolidated Standards of Reporting Trials
CRTs: Cluster Randomized Controlled Trials
CTU: Clinical Trials Units
EQUATOR: Enhancing the QUAlity and Transparency Of health Research
LMICs: Lower-middle-income countries
PPI: Patient and Public Involvement
SAPs: Statistical analysis plans
SSA: Statistical Society of Australia
SW-CRTs: Stepped wedge cluster randomized controlled trials
UKCRC CTUs: UK Clinical Research Collaboration registered Clinical Trial Units
